# Urate-Lowering Therapy May Prevent the Development of Coronary Artery Disease in Patients With Gout

**DOI:** 10.3389/fmed.2020.00063

**Published:** 2020-02-27

**Authors:** Fu-Shun Yen, Chih-Cheng Hsu, Hsin-Lun Li, James Cheng-Chung Wei, Chii-Min Hwu

**Affiliations:** ^1^Dr. Yen's Clinic, Taoyuan City, Taiwan; ^2^Institute of Population Health Sciences, National Health Research Institutes, Miaoli County, Taiwan; ^3^Department of Health Services Administration, China Medical University, Taichung City, Taiwan; ^4^Department of Family Medicine, Min-Sheng General Hospital, Taoyuan City, Taiwan; ^5^Management Office for Health Data, China Medical University Hospital, Taichung City, Taiwan; ^6^Graduate Institute of Clinical Medical Science, College of Medicine, China Medical University, Taichung City, Taiwan; ^7^Department of Rheumatology, BenQ Medical Center, The Affiliated BenQ Hospital of Nanjing Medical University, Nanjing, China; ^8^Institute of Medicine, Chung Shan Medical University, Taichung City, Taiwan; ^9^Department of Medicine, Chung Shan Medical University Hospital, Taichung City, Taiwan; ^10^Graduate Institute of Integrated Medicine, China Medical University, Taichung City, Taiwan; ^11^Faculty of Medicine, National Yang-Ming University School of Medicine, Taipei, Taiwan; ^12^Section of Endocrinology and Metabolism, Department of Medicine, Taipei Veterans General Hospital, Taipei, Taiwan

**Keywords:** urate-lowering therapy, coronary artery disease, stroke, heart failure, gout

## Abstract

Substantial evidence has demonstrated a close relationship between hyperuricemia and cardiovascular (CV) diseases, but few studies have explored the possibility of using urate-lowering therapy (ULT) to attenuate the development of CV diseases. To compare the risks of incident coronary artery disease (CAD), stroke, and heart failure (HF) between ULT users and non-users in patients with gout, we conducted a retrospective cohort study from the population-based National Health Insurance Research Database in Taiwan. In total, 4,072 patients with gout were included between 2000 and 2012. The overall incident rates of CAD, stroke, and HF were compared between 2,036 ULT users and 2,036 matched non-users. The incident rates of incident CAD were 1.3 and 1.7 per 100 person-years for ULT users and non-users. ULT users had a lower adjusted hazard ratio (aHR) for CAD [aHR: 0.7, 95% confidence interval (CI): 0.55–0.89] compared with non-users. ULT users also had a lower aHR for incident stroke (aHR: 0.68, 95% CI: 0.5–0.92) compared with non-users. ULT had a neutral effect on the risk of incident HF (aHR: 0.92, 95% CI: 0.58–1.45). Among the urate-lowering therapy, subgroup analyses indicated that uricosuric agents had a significant effect on the prevention of CAD and stroke development; and the protection against the development of CAD by uricosuric agents appeared to have a dose-response trend. Our study demonstrated that ULT associated with lower risks of incident CAD and stroke. We recommend that patients with gout receive ULT to lower the burden of CV diseases.

## Introduction

Cardiovascular (CV) disease is the leading cause of morbidity and mortality worldwide (1) and accounts for ~20% of overall deaths in Taiwan (2). Among CV diseases, coronary artery disease (CAD) is the primary cause of death in developed countries (2). Stroke is also a major cause of death, and patients who had a stroke live with long-term disabilities (3), which creates a considerable burden in terms of care and costs on families and the state (4). After several decades of studies and advanced work on this topic, the incidences of CAD and stroke were reported to have prominently decreased, but the occurrence of heart failure (HF) did not widely vary (5). The management of HF progressed, but HF-induced mortality remains high (6).

Patients with gout have a higher risk of CV diseases compared with patients without gout (7). Whether this risk is due to inflammation in gout or hyperuricemia remains unclear. A large body of evidence supports the close relationship between serum uric acid (UA) and CV diseases. A meta-analysis disclosed an elevated CAD risk in individuals with hyperuricemia (8). Another meta-analysis revealed that hyperuricemia was associated with a high risk of stroke (9). Hyperuricemia was also associated with a significantly higher risk of new-onset HF, showing a dose-response effect (10). However, this association does not attest to causation; indeed, hyperuricemia always appears with CV diseases. Using urate-lowering therapy (ULT) might help clarify the relationship between serum UA and CV diseases. If ULT attenuates the development of CV diseases, the global burden of the disease would be prominently reduced. However, research on ULT in the development of CV diseases is scant; therefore, we conducted this retrospective cohort study to assess the possibility of using ULT to reduce the risks of new-onset CAD, stroke, and HF.

## Materials and Methods

### Data Source

In 1995, Taiwan implemented a single-payer National Health Insurance (NHI) program providing citizens with a universal and comprehensive health care system. Approximately 99.5% of the population is covered by the NHI program. The recorded medical utilizations are collected by the NHI Research Database (NHIRD). The database contains information on outpatient visits, inpatient care, medical procedures, and medications. The International Classification of Diseases, 9th Revision, Clinical Modification (ICD-9-CM) codes are used for disease coding (11). One million patients were randomly selected from the NHIRD in 2000 to form a study database named NHIRD 2000, which was used in our study. The Research Ethics Committee of China Medical University and Hospital approved this study (CMUH-104-REC2-115). Because identifiable information in the NHIRD is encrypted, informed consent was exempted by the research ethics committee.

### Patients Selection and Endpoints

Patients with gouty arthritis (ICD-9-CM: 274.00–274.03 and 274.8–274.9) aged between 20 and 79 years from 2000 to 2012 were identified from the NHIRD 2000. The case cohort included patients who had received ULT with allopurinol, febuxostat, benzbromarone, sulfinpyrazone, and probenecid. The control cohort included patients with a diagnosis of gouty arthritis who were never prescribed urate-lowering medication during the follow-up period. Main outcomes were incident CAD (410–414), stroke (362.34, 430.x−438.x), and HF (428). Any patient diagnosed as having CAD, stroke, or HF before or half a year after the index date; who had received chemotherapy or been diagnosed as having cancer by the index date; or who had missing age, sex, or region data was excluded. The index date for the case cohort was set as the date of receiving first ULT. For the control cohort, a date was randomly assigned from within the observation period. The follow-up period for CAD, stroke, and HF ended in the event of individual outcome, death, withdrawal from the NHI program, or at the end of 2013.

### Statistical Analysis

Using a logistic regression model, we applied the propensity-score matching method to age, sex, residential area, index year, and year of gouty arthritis diagnosis between cases and controls in a 1:1 ratio (12). The standardized mean difference (SMD) was applied on the strata of age, sex, area, comorbidity, medication, and follow-up period to verify the comparability between these two groups. We balanced the frequency distribution of the case and control cohorts on sex, age, residential area, comorbidity [i.e., hypertension (HT: 401–405 and A26), diabetes mellitus (DM: 250.x0, 250.x2, and A181), hypercholesterolemia (272, 278, and A189), peripheral vascular diseases (093.0, 437.3, 440.x, 441.x, 443.1–443.9, 47.1, 557.1, 557.9, and V43.4), atrial fibrillation (427.3), rheumatologic diseases (446.5, 710.0–710.4, 714.0–714.2, 714.8, and 725.x), renal diseases (403.01, 403.11, 403.91, 404.02, 404.03, 404.12, 404.13, 404.92, 404.93, V42.0, V45.1, V56.x, and 790), and alcohol-related diseases (291.x, 303.x, 305.0, 357.5, 425.5, 535.3, 571.0–571.3, 980.0, and E947.3)], and medications [i.e., angiotensin-converting enzyme (ACE) inhibitors, angiotensin II receptor blockers (ARBs), β-blockers, calcium channel blockers, diuretics, potassium-sparing diuretics, metformin, sulfonylureas, insulin, statin, and aspirin]. The incidence rate of events was defined as the number of events divided by the observed person-years. We performed subgroup analyses dividing xanthine oxidase inhibitors, uricosuric agents, basic demographics, comorbidity and medications to see their different influence on the risks of incident CAD and stroke. To assess the dose–response relationship, we analyzed the risks of CAD and stroke development by three equally distributed cumulative durations of uricosuric agent treatments (≤1, 1–5, or >5 months) and the cumulative mean defined daily dose (DDD) of therapy (≤0.5, 0.5–0.8, or >0.8 DDD/month) relative to no-use of ULT. We tried to find out which intervals of ULT duration was more evenly patients distributed, and the intervals of ULT duration (0–1/1–5/>5 months) was determined. The cumulative mean DDD was calculated by dividing the cumulative DDD by the period of uricosuric agent use. The WHO set the DDD at 100 mg for benzbromarone, 1 g for probenecid, 300 mg for sulfinpyrazone, 400 mg for allopurinol, and 80 mg for febuxostat. Crude hazard ratios (cHRs), adjusted HRs (aHRs), and 95% confidence intervals (CIs) were derived from multivariate Cox proportional hazards regression models. The Kaplan–Meier method was used to obtain the cumulative incidences of CAD, stroke, and HF in patients who received ULT and those who did not. We used the log-rank test to detect their statistical significance. All analyses were performed using SAS 9.4 software program (SAS Institute, Cary, NC, USA). The null hypothesis of no effective difference between the two groups was rejected if *P* < 0.05.

## Results

**Figure 1:**
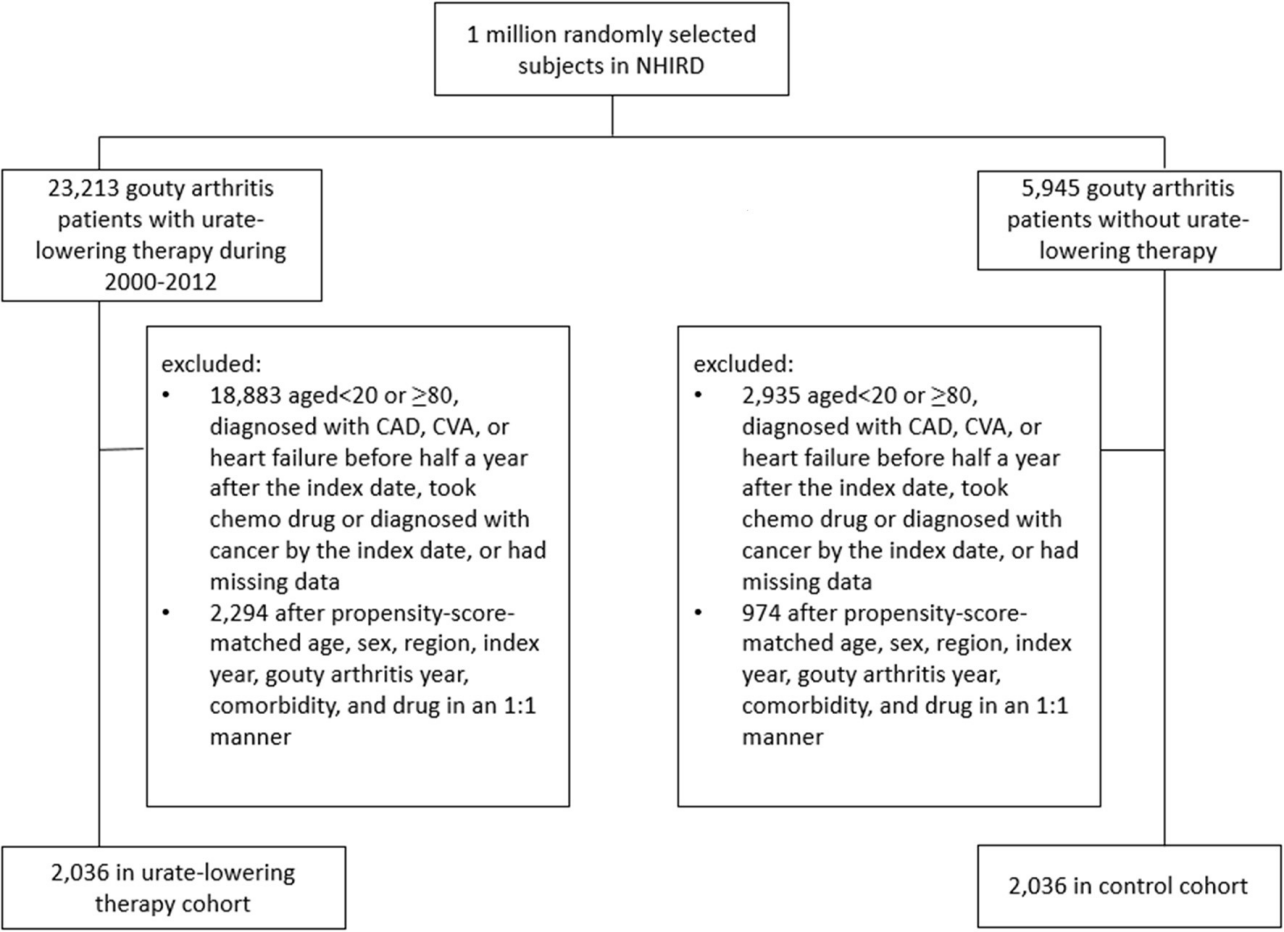
Flow chart of the process for deriving the urate-lowering therapy cohort and the control cohort from the National Health Insurance Research Database.

Figure 1 illustrates the flow chart for the selection of the ULT case cohort and non-ULT control cohort from the NHIRD. After matching, most variables were balanced except for ULT users who had a slightly longer follow-up period than did non-users (SMD = 0.13). The mean (standard deviation, SD) age of ULT users and non-users was 44.9 (13) and 45.1 (13.6) years (Table 1). The mean (SD) follow-up period of ULT users and non-users was 4.55 (2.86) and 4.18 (2.8) years, respectively.

**Table 1:** Baseline characteristics in the study cohorts receiving or not receiving urate-lowering therapy.

	**Before matching Urate-lowering therapy**					**After matching Urate-lowering therapy**				
	**No**		**Yes**		**SMD**	**No**		**Yes**		**SMD**
	**3,010**		**4,330**			**2,036**		**2,036**		
	***n***	**%**	***n***	**%**		***n***	**%**	***n***	**%**	
**SEX**										
Male	2,285	75.9	3918	90.5	0.41	1,721	84.5	1,734	85.2	0.02
**AGE, YEARS**										
20–39	1,100	36.5	2,053	47.4	0.22	807	39.6	843	41.4	0.04
40–59	1,403	46.6	1,711	39.5	0.14	930	45.7	876	43	0.05
60–79	507	16.8	566	13.1	0.11	299	14.7	317	15.6	0.02
Mean (SD)	46.2	13.7	43	13.9	0.23	45.1	13.6	44.9	14	0.01
**AREA**										
North	1,412	46.9	2,028	46.8	0.001	951	46.7	915	44.9	0.04
Central	627	20.8	931	21.5	0.02	434	21.3	418	20.5	0.02
South	872	29	1207	27.9	0.02	580	28.5	623	30.6	0.05
Other	99	3.3	164	3.8	0.03	71	3.5	80	3.9	0.02
**COMORBIDITY**										
Hypertension	612	20.3	919	21.2	0.02	436	21.4	412	20.2	0.03
DM	223	7.4	195	4.5	0.13	132	6.5	117	5.7	0.03
Hypercholesterolemia	643	21.4	930	21.5	0.003	437	21.5	419	20.6	0.02
Peripheral vascular diseases	56	1.9	44	1	0.07	29	1.4	27	1.3	0.01
Atrial fibrillation	5	0.2	6	0.1	0.01	2	0.1	2	0.1	<0.001
Rheumatologic diseases	98	3.3	59	1.4	0.13	39	1.9	41	2	0.01
Renal diseases	66	2.2	111	2.6	0.02	41	2	48	2.4	0.02
Alcohol-related diseases	89	3	123	2.8	0.01	65	3.2	67	3.3	0.01
CCI(SD)	0.16	0.56	0.09	0.42	0.14	0.12	0.5	0.12	0.49	0.002
**DRUG**										
ACE inhibitors/ARBs	345	11.5	509	11.8	0.01	245	12	230	11.3	0.02
β-blockers	713	23.7	880	20.3	0.08	457	22.4	453	22.2	0.005
Calcium-channel blockers	533	17.7	714	16.5	0.03	361	17.7	337	16.6	0.03
Diuretics	503	16.7	619	14.3	0.07	312	15.3	321	15.8	0.01
Potassium sparing diuretics	56	1.9	32	0.7	0.11	27	1.3	29	1.4	0.01
Other antihypertensive	227	7.5	329	7.6	0.002	155	7.6	153	7.5	0.004
Metformin	129	4.3	134	3.1	0.06	86	4.2	72	3.5	0.04
sulfonylurea	138	4.6	152	3.5	0.06	87	4.3	81	4	0.01
Insulin	33	1.1	50	1.2	0.01	30	1.5	22	1.1	0.03
Statin	176	5.8	153	3.5	0.11	101	5	99	4.9	0.005
Aspirin	250	8.3	299	6.9	0.05	162	8	163	8	0.002
mean of follow-up period of outcome (years)	3.61	2.6	6.56	3.66	0.9	4.18	2.8	4.55	2.86	0.13

*SMD, standardized mean difference, ≤0.10 indicates a negligible difference between the two cohorts; outcomes consisting of coronary artery disease, stroke, and heart failure. ACE inhibitors, angiotensin-converting enzyme inhibitors; ARBs, angiotensin II receptor blockers*.

**Figure 2:**
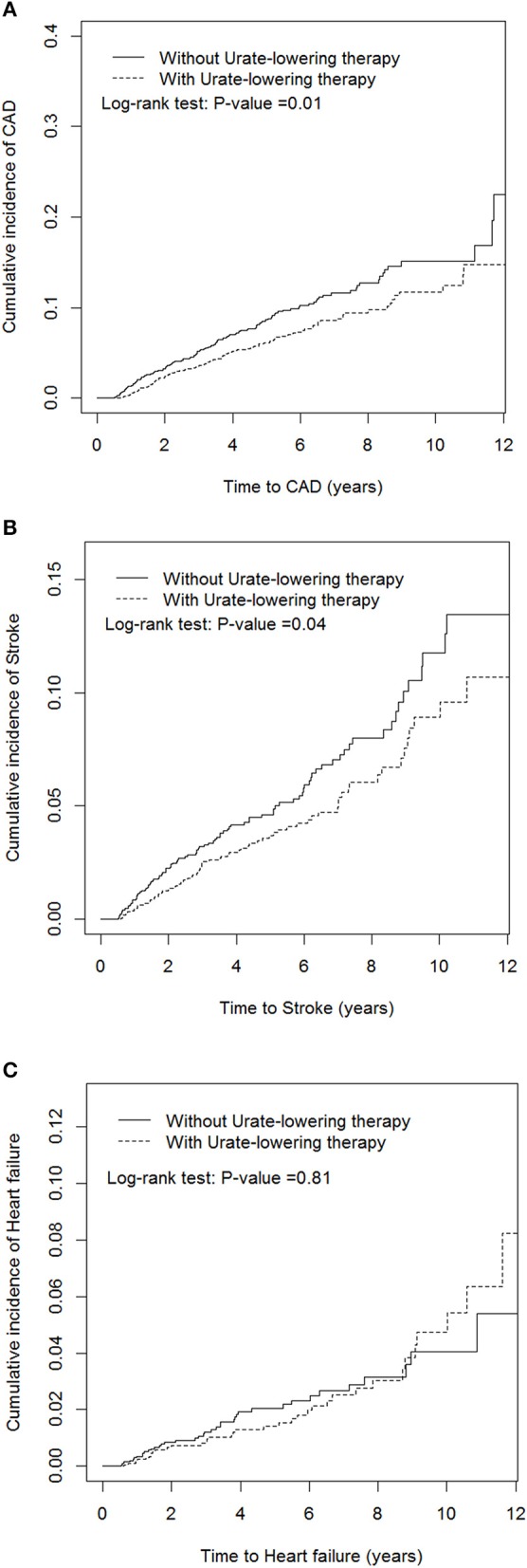
Cumulative incidences of new-onset coronary artery disease **(A)**, stroke **(B)**, and heart failure **(C)** in patients receiving and not receiving urate-lowering therapy.

Table 2 indicates that patients receiving ULT were 30% less likely to develop CAD than non-users (aHR = 0.7, *P* = 0.004). Patients receiving ULT were 32% less likely to develop stroke than non-users (aHR = 0.68, *P* = 0.01). ULT users were less likely to develop ischemic stroke (aHR = 0.59, *P* = 0.02), but ULT had no significant effect on the development of hemorrhagic stroke. ULT had a neutral effect on the development of HF (aHR = 0.92, *P* = 0.72). Figure 2 illustrates the cumulative incidences of incident CAD and stroke in patients who received ULT and those who did not, using the Kaplan–Meier method.

**Table 2:** Incident rates of coronary artery disease, stroke, and heart failure between urate-lowering drug users vs. non-users after propensity matching.

**Outcome**	**Urate-lowering therapy**						**cHR (95%CI)**	***P***	**aHR (95%CI)**	***P***
	**No**			**Yes**						
	**Event**	**PY**	**IR**	**Event**	**PY**	**IR**				
CAD	155	8,882	1.7	123	9,506	1.3	0.73 (0.58, 0.93)	0.01	0.7 (0.55, 0.89)	0.004
Stroke	100	9,137	1.1	78	9,657	0.8	0.73 (0.55, 0.99)	0.04	0.68 (0.5, 0.92)	0.01
Ischemic stroke	54	9,137	0.6	35	9,657	0.4	0.61 (0.4, 0.93)	0.02	0.59 (0.38, 0.91)	0.02
Hemorrhagic stroke	14	9,137	0.2	9	9,657	0.1	0.6 (0.26, 1.4)	0.24	0.52 (0.22, 1.23)	0.14
Heart failure	39	9,364	0.4	39	9,792	0.4	0.95 (0.61, 1.48)	0.81	0.92 (0.58, 1.45)	0.72

*IR, incidence rate, per 100 person-years; CI, confidence interval; P, P-value; cHR, crude hazard ratio; aHR, adjusted hazard ratio, controlling for sex, age, area, every comorbidity, and drug in Table 1; CAD, coronary artery disease*.

Table 3 discloses that ULT users, as compared with non-users, have significantly lower risk of CAD, especially in patients taking uricosuric agents, age 40–59 years, patients with HT or hypercholesterolemia; patients without DM, peripheral vascular diseases, atrial fibrillation, rheumatologic diseases, renal diseases, alcohol-related diseases; patients without taking anti-hypertensive drugs, anti-diabetic agents, statin or aspirin. Table 4 discloses that ULT users, as compared with non-users, have significantly lower risk of stroke, especially in patients taking uricosuric agents, male, age 40–59 years, patients with HT or alcohol-related diseases; patients without hypercholesterolemia, peripheral vascular disease, atrial fibrillation, rheumatologic diseases, renal diseases, or alcohol-related diseases; patients taking anti-hypertensive drugs, or statin; patients without taking anti-diabetic agents, statin or aspirin.

**Table 3:** Incidence and hazard ratio of CAD between cohorts receiving or not receiving urate-lowering therapy.

		**Urate-lowering therapy**							
		**No**			**Yes**				
		**Event**	**PY**	**IR**	**Event**	**PY**	**IR**	**cHR (95%CI)**	**aHR (95%CI)**
Overall		155	8,882	1.7	123	9,506	1.3	0.73 (0.58, 0.93)^**^	0.7 (0.55, 0.89)^**^
XO inhibitors		155	8,882	1.7	42	2,811	1.5	0.85 (0.6, 1.19)	0.78 (0.55, 1.11)
Uricosuric agents		155	8,882	1.7	81	6,695	1.2	0.68 (0.52, 0.9)^**^	0.67 (0.51, 0.87)^**^
**SEX**									
Female		47	1,633	2.9	41	1,716	2.4	0.82 (0.54, 1.24)	0.67 (0.43, 1.07)
Male		108	7,249	1.5	82	7,790	1.1	0.7 (0.53, 0.93)^*^	0.77 (0.57, 1.02)
**AGE, YEARS**									
20–39		16	3,679	0.4	14	3,989	0.4	0.78 (0.38, 1.61)	0.81 (0.38, 1.74)
40–59		98	3,930	2.5	71	4,043	1.8	0.68 (0.5, 0.93)^*^	0.68 (0.5, 0.93)^*^
60–79		41	1,274	3.2	38	1,474	2.6	0.8 (0.51, 1.25)	0.74 (0.46, 1.19)
**COMORBIDITY**									
Hypertension	No	89	7,221	1.2	73	7,679	1.0	0.76 (0.56, 1.04)	0.78 (0.57, 1.07)
	Yes	66	1,662	4.0	50	1,828	2.7	0.68 (0.47, 0.98)^*^	0.64 (0.44, 0.94)^*^
DM	No	133	8,316	1.6	102	8,998	1.1	0.7 (0.54, 0.91)^**^	0.66 (0.51, 0.86)^**^
	Yes	22	566	3.9	21	508	4.1	1.12 (0.61, 2.06)	1.15 (0.55, 2.38)
Hypercholesterolemia	No	103	7,151	1.4	89	7,601	1.2	0.8 (0.6, 1.07)	0.77 (0.58, 1.03)
	Yes	52	1731	3.0	34	1,905	1.8	0.58 (0.37, 0.89)^*^	0.55 (0.35, 0.86)^**^
Peripheral vascular diseases	No	151	8,736	1.7	120	9,371	1.3	0.73 (0.57, 0.93)^*^	0.7 (0.55, 0.89)^**^
	Yes	4	146	2.7	3	136	2.2	0.76 (0.17, 3.43)	
Atrial fibrillation	No	154	8,878	1.7	123	9,495	1.3	0.74 (0.58, 0.93)^*^	0.71 (0.56, 0.9)^**^
	Yes	1	4	25	0	11	0		
Rheumatologic diseases	No	149	8717	1.7	121	9281	1.3	0.75 (0.59, 0.96)^*^	0.72 (0.57, 0.92)^**^
	Yes	6	165	3.6	2	226	0.9	0.27 (0.05, 1.33)	0.15 (0.02, 1.4)
Renal diseases	No	151	8,745	1.7	121	9,293	1.3	0.75 (0.59, 0.95)^*^	0.72 (0.57, 0.92)^**^
	Yes	4	137	2.9	2	213	0.9	0.34 (0.06, 1.88)	
Alcohol-related diseases	No	152	8,680	1.8	123	9,222	1.3	0.75 (0.59, 0.95)^*^	0.72 (0.56, 0.91)^**^
	Yes	3	203	1.5	0	284	0		
**DRUG**									
ACE inhibitors/ARBs	No	117	8,052	1.5	92	8,502	1.1	0.73 (0.56, 0.97)^*^	0.73 (0.55, 0.96)^*^
	Yes	38	830	4.6	31	1,004	3.1	0.66 (0.41, 1.07)	0.65 (0.39, 1.08)
β-blockers	No	98	7,092	1.4	72	7,453	1.0	0.69 (0.51, 0.94)^*^	0.73 (0.54, 0.99)^*^
	Yes	57	1,790	3.2	51	2,053	2.5	0.78 (0.53, 1.13)	0.7 (0.47, 1.03)
Calcium-channel blockers	No	102	7,583	1.3	79	8,068	1.0	0.72 (0.54, 0.96)^*^	0.73 (0.54, 0.98)^*^
	Yes	53	1,299	4.1	44	1,438	3.1	0.74 (0.5, 1.11)	0.69 (0.45, 1.06)
Diuretics	No	116	7,690	1.5	86	7,936	1.1	0.71 (0.54, 0.94)^*^	0.72 (0.54, 0.96)^*^
	Yes	39	1,193	3.3	37	1,570	2.4	0.72 (0.46, 1.13)	0.71 (0.44, 1.13)
Potassium sparing diuretics	No	153	8,799	1.7	120	9,377	1.3	0.73 (0.57, 0.92)^**^	0.69 (0.54, 0.88)^**^
	Yes	2	83	2.4	3	129	2.3	0.98 (0.16, 6.08)	
Metformin	No	146	8,573	1.7	109	9,209	1.2	0.69 (0.54, 0.88)^**^	0.65 (0.51, 0.84)^***^
	Yes	9	309	2.9	14	297	4.7	1.58 (0.68, 3.69)	1.49 (0.56, 3.97)
Sulfonylurea	No	144	8,535	1.7	107	9,153	1.2	0.68 (0.53, 0.88)^**^	0.65 (0.5, 0.83)^***^
	Yes	11	347	3.2	16	354	4.5	1.44 (0.67, 3.12)	1.55 (0.63, 3.85)
Insulin	No	153	8,785	1.7	118	9,439	1.3	0.71 (0.56, 0.9)^**^	0.67 (0.53, 0.86)^**^
	Yes	2	97	2.1	5	67	7.4	3.06 (0.59, 15.93)	
Statin	No	146	8,556	1.7	110	9,076	1.2	0.7 (0.55, 0.9)^**^	0.68 (0.53, 0.87)^**^
	Yes	9	326	2.8	13	430	3.0	1.09 (0.46, 2.57)	1.03 (0.39, 2.7)
Aspirin	No	139	8,253	1.7	104	8,794	1.2	0.69 (0.54, 0.89)^**^	0.67 (0.52, 0.87)^**^
	Yes	16	629	2.5	19	713	2.7	1.03 (0.53, 2)	0.92 (0.45, 1.87)

IR, incidence rate, per 100 person-years; PY, person-years; CI, confidence interval; cHR, crude hazard ratio; aHR, adjusted hazard ratio, controlling for sex, age, area, and every comorbidity, and drug in Table 2; XO inhibitors, xanthine oxidase inhibitors, consisting of allopurinol and febuxostat; Uricosuric agents, consisting of benzbromarone, probenecid, and sulfinpyrazone; ACE inhibitors, angiotensin-converting enzyme inhibitors; ARBs, angiotensin II receptor blockers;

* P < 0.05,

** P < 0.01, and

*** *P < 0.001*.

**Table 4:** Incidence and hazard ratio of stroke between cohorts receiving or not receiving urate-lowering therapy.

		**Urate-lowering therapy**							
	**No**			**Yes**					
	**Event**	**PY**	**IR**	**Event**	**PY**	**IR**	**cHR (95%CI)**	**aHR (95%CI)**	
**Overall**	100	9,137	1.1	78	9,657	0.8	0.73 (0.55, 0.99)^*^	0.68 (0.5, 0.92)^*^	
XO inhibitors	100	9,137	1.1	27	2,873	0.9	0.86 (0.56, 1.31)	0.76 (0.49, 1.18)	
Uricosuric agents	100	9,137	1.1	51	6,785	0.8	0.68 (0.49, 0.96)^*^	0.65 (0.46, 0.91)^*^	
**SEX**									
Female		20	1,767	1.1	26	1,802	1.4	1.26 (0.71, 2.26)	0.64 (0.33, 1.26)
Male		80	7,370	1.1	52	7,855	0.7	0.61 (0.43, 0.86)^**^	0.64 (0.45, 0.91)^*^
**AGE, YEARS**									
20–39		10	3,720	0.3	9	4,000	0.2	0.83 (0.34, 2.04)	1.08 (0.39, 3.04)
40–59		48	4,147	1.2	33	4,147	0.8	0.68 (0.44, 1.06)	0.62 (0.39, 0.97)^*^
60–79		42	1,270	3.3	36	1,510	2.4	0.7 (0.45, 1.1)	0.7 (0.43, 1.13)
**COMORBIDITY**									
Hypertension	No	49	7,443	0.7	43	7,770	0.6	0.84 (0.56, 1.26)	0.86 (0.56, 1.3)
	Yes	51	1,693	3.0	35	1,887	1.9	0.59 (0.39, 0.91)^*^	0.48 (0.3, 0.77)^**^
DM	No	82	8,589	1.0	66	9,110	0.7	0.76 (0.55, 1.04)	0.72 (0.52, 1.01)
	Yes	18	548	3.3	12	547	2.2	0.68 (0.33, 1.42)	0.43 (0.18, 1.01)
Hypercholesterolemia	No	66	7,361	0.9	51	7,730	0.7	0.73 (0.51, 1.06)	0.68 (0.47, 0.99)^*^
	Yes	34	1,776	1.9	27	1,927	1.4	0.68 (0.41, 1.13)	0.64 (0.37, 1.1)
Peripheral vascular diseases	No	94	9,002	1.0	74	9,528	0.8	0.74 (0.55, 1)	0.68 (0.5, 0.93)^*^
	Yes	6	135	4.5	4	129	3.1	0.66 (0.19, 2.38)	
Atrial fibrillation	No	100	9,125	1.1	77	9,651	0.8	0.72 (0.54, 0.97)^*^	0.67 (0.49, 0.91)^**^
	Yes	0	12	0	1	7	15		
Rheumatologic diseases	No	98	8,937	1.1	77	9,424	0.8	0.74 (0.55, 1)^*^	0.69 (0.51, 0.94)^*^
	Yes	2	200	1.0	1	233	0.4	0.42 (0.04, 4.66)	
Renal diseases	No	97	8,991	1.1	76	9,454	0.8	0.74 (0.55, 1)	0.7 (0.51, 0.95)^*^
	Yes	3	146	2.1	2	203	1.0	0.48 (0.08, 2.87)	
Alcohol-related diseases	No	94	8,937	1.1	74	9,390	0.8	0.74 (0.55, 1.01)	0.7 (0.52, 0.96)^*^
	Yes	6	199	3.0	4	267	1.5	0.5 (0.14, 1.78)	0.12 (0.02, 0.88)^*^
**DRUG**									
ACE inhibitors/ARBs	No	70	8,295	0.8	60	8,605	0.7	0.82 (0.58, 1.16)	0.85 (0.6, 1.21)
	Yes	30	841	3.6	18	1052	1.7	0.47 (0.26, 0.85)^*^	0.25 (0.13, 0.49)^***^
β-blockers	No	56	7,260	0.8	48	7,519	0.6	0.82 (0.56, 1.21)	0.84 (0.56, 1.24)
	Yes	44	1,877	2.3	30	2,138	1.4	0.59 (0.37, 0.94)^*^	0.41 (0.25, 0.69)^***^
Calcium-channel blockers	No	54	7,808	0.7	54	8,144	0.7	0.96 (0.66, 1.4)	1.02 (0.69, 1.5)
	Yes	46	1,329	3.5	24	1,514	1.6	0.44 (0.27, 0.72)^**^	0.34 (0.2, 0.58)^***^
Diuretics	No	70	7,886	0.9	52	8,057	0.7	0.73 (0.51, 1.04)	0.71 (0.49, 1.02)
	Yes	30	1,251	2.4	26	1,601	1.6	0.69 (0.41, 1.18)	0.55 (0.31, 0.96)^*^
Potassium sparing diuretics	No	100	9,053	1.1	75	9,521	0.8	0.71 (0.53, 0.96)^*^	0.66 (0.48, 0.89)^**^
	Yes	0	84	0.0	3	136	2.2		
Metformin	No	90	8,852	1.0	71	9,347	0.8	0.74 (0.55, 1.02)	0.7 (0.51, 0.96)^*^
	Yes	10	285	3.5	7	311	2.3	0.58 (0.21, 1.59)	0.46 (0.13, 1.62)
sulfonylurea	No	92	8,805	1.0	71	9,289	0.8	0.73 (0.53, 0.99)^*^	0.68 (0.5, 0.94)^*^
	Yes	8	331	2.4	7	369	1.9	0.86 (0.31, 2.37)	0.56 (0.15, 2.05)
Insulin	No	98	9,038	1.1	76	9,584	0.8	0.73 (0.54, 0.98)^*^	0.66 (0.49, 0.9)^**^
	Yes	2	98	2.0	2	73	2.7	1.22 (0.17, 8.69)	
Statin	No	92	8,802	1.0	72	9,215	0.8	0.74 (0.55, 1.01)	0.71 (0.52, 0.98)^*^
	Yes	8	334	2.4	6	442	1.4	0.5 (0.17, 1.46)	0.14 (0.03, 0.69)^*^
Aspirin	No	86	8,502	1.0	69	8,919	0.8	0.76 (0.55, 1.04)	0.71 (0.52, 0.99)^*^
	Yes	14	635	2.2	9	738	1.2	0.55 (0.24, 1.27)	0.43 (0.16, 1.19)

*IR, incidence rate, per 100 person-years; PY, person-years; CI, confidence interval; cHR, crude hazard ratio; aHR, adjusted hazard ratio, controlling for sex, age, area, every comorbidity, and drug in Table 1; XO inhibitors, xanthine oxidase inhibitors, consisting of allopurinol, and febuxostat; Uricosuric agents, consisting of benzbromarone, probenecid, and sulfinpyrazone; ACE inhibitors, angiotensin-converting enzyme inhibitors*.

ARBs, angiotensin II receptor blockers;

* P < 0.05,

** P < 0.01, and

*** *P < 0.001*.

Table 5 depicts that uricosuric agent users with a cumulative duration of therapy ≤1, 1–5, and >5 months were 0.82, 0.88, and 0.44 times less likely to develop CAD compared with non-users (*P* for trend < 0.001). Uricosuric agent users with a cumulative mean DDD (per month) of ≤0.5, 0.5–0.8, and >0.8 were 0.62, 0.55, and 0.74 times less likely to develop CAD than non-users (*P* for trend = 0.02). Table 6 indicates that uricosuric agent users with a cumulative duration of therapy of ≤1, 1–5, and >5 months were 0.71, 0.87, and 0.43 times less likely to develop stroke than non-users (*P* for trend = 0.003). Uricosuric agent users with a cumulative mean DDD (per month) of ≤0.5, 0.5–0.8, and >0.8 were 0.54, 0.42, and 0.84 times less likely to develop stroke than non-users (*P* for trend = 0.1).

**Table 5:** Cox model measured hazard ratios and 95% confidence interval of coronary artery disease associated with sex, age, and comorbidity restricted on uricosuric agents after propensity-score matching.

**Variable**	**Event**	**PY**	**IR**	**cHR (95%CI)**	**aHR (95%CI)**
**MATCHED COHORT**					
Non-users (*n* = 2,036)	155	8,882	1.7	1 (ref)	1 (ref)
Users (*n* = 1,454)	81	6,695	1.2	0.68 (0.52,0.9)^**^	0.67 (0.51,0.87)^**^
**CUMULATIVE DURATION OF THERAPY (MONTHS)**					
Non-users	155	8,882	1.7	1 (ref)	1 (ref)
≦1	22	1,708	1.3	0.72 (0.46, 1.12)	0.82 (0.52, 1.29)
1-5	33	2,204	1.5	0.86 (0.59, 1.25)	0.88 (0.6, 1.28)
>5	26	2,784	0.9	0.52 (0.35, 0.8)^**^	0.44 (0.28, 0.67)^***^
*P* for trend				0.003	<0.001
**MONTHLY AVERAGE DAILY DOSES OF URATE-LOWERING THERAPY**					
Non-users	155	8,882	1.7	1 (ref)	1 (ref)
≦0.5	26	2,139	1.2	0.68 (0.45, 1.04)	0.62 (0.41, 0.94)^*^
0.5–0.8	15	1,524	1.0	0.56 (0.33, 0.95)^*^	0.55 (0.32, 0.94)^*^
>0.8	40	3,032	1.3	0.74 (0.52, 1.05)	0.74 (0.52, 1.06)
*P* for trend				0.02	0.02

IR, incidence rate, per 100 person-years; PY, person-years; CI, confidence interval; cHR, crude hazard ratio; aHR, adjusted hazard ratio, controlling for sex, age, area, every comorbidity, and drug in Table 2;

* P < 0.05,

** P < 0.01, and

*** *P < 0.001*.

**Table 6:** Cox model measured hazard ratios and 95% confidence interval of stroke associated with sex, age, and comorbidity restricted on uricosuric agents after propensity-score matching.

**Variable**	**Event**	**PY**	**IR**	**cHR (95%CI)**	**aHR (95%CI)**
**MATCHED COHORT**					
Non-users (*n* = 2,036)	100	9,137	1.1	1 (ref)	1 (ref)
Users (*n* = 1,454)	51	6,785	0.8	0.68 (0.49, 0.96)^*^	0.65 (0.46, 0.91)^*^
**CUMULATIVE DURATION OF THERAPY (MONTHS)**					
Non-users	100	9,137	1.1	1 (ref)	1 (ref)
≦1	12	1,737	0.7	0.64 (0.35, 1.17)	0.71 (0.39, 1.32)
1–5	22	2,228	1.0	0.9 (0.57, 1.44)	0.87 (0.54, 1.4)
>5	17	2,820	0.6	0.54 (0.32, 0.9)^*^	0.43 (0.26, 0.73)^**^
*P* for trend				0.03	0.003
**MONTHLY AVERAGE DAILY DOSES OF URATE-LOWERING THERAPY**					
Non-users	100	9,137	1.1	1 (ref)	1 (ref)
≦0.5	16	2,179	0.7	0.67 (0.39, 1.13)	0.54 (0.32, 0.93)^*^
0.5–0.8	8	1,537	0.5	0.47 (0.23, 0.96)^*^	0.42 (0.2, 0.87)^*^
>0.8	27	3,069	0.9	0.8 (0.53, 1.23)	0.84 (0.54, 1.29)
*P* for trend				0.1	0.1

IR, incidence rate, per 100 person-years; PY, person-years; CI, confidence interval; cHR, crude hazard ratio; aHR, adjusted hazard ratio, controlling for sex, age, area, every comorbidity, and drug in Table 2;

* P < 0.05,

** P < 0.01, and

^***^P < 0.001.

## Discussion

The results of this study demonstrated that ULT in patients with gout was associated with lower risks of new-onset CAD and stroke. Subgroup analyses revealed that uricosuric agents had a significant effect on the reduction of incident CAD and stroke. The effect of uricosuric agents on the reduction of CAD appeared to follow a dose-response relationship.

Some underlying mechanisms might be responsible for the association between serum UA and CV diseases. (1) An animal study demonstrated that hyperuricemia could stimulate the renin-angiotensin system, inhibit the release of endothelial nitric oxide, and increase blood pressure (14). (2) A body of evidence associated UA with the metabolic syndromes of HT, obesity, dyslipidemia, and insulin resistance (13), which are all precursors of CV disease. (3) Hyperuricemia is associated with endothelial cell dysfunction, endothelial nitric oxide synthase uncoupling, and elevated reactive oxygen species, which can accelerate vascular cell apoptosis and promote atherosclerosis (15). (4) In cultured rat aortic cells, UA could induce vascular smooth muscle cell proliferation through the mitogen-activated protein kinase pathway and platelet-derived growth factor receptor-β phosphorylation (16). (5) UA can increase the blood C-reactive protein level and the circulating nuclear factor-κ B, interleukin-1β, interleukin 6, and tumor necrosis factor α; these proinflammatory effects of UA can strengthen its proatherogenic properties (15, 17). (6) Serum UA was reported to enhance the oxygenation of low-density lipoprotein cholesterol and to facilitate lipid peroxidation (7). (7) Hyperuricemia might increase platelet adhesiveness and aggregation, which could potentiate thrombus formation (18). Moreover, the human atherosclerotic plaque contains a considerable amount of UA (19).

Wheeler et al. (8) conducted a meta-analysis of 16 prospective studies and discovered that a high level of UA increased odds ratio by 1.13 for incident CAD in the general population; however, the odds ratio decreased to 1.02 after adjustment for other risk factors. Kim et al. (20) conducted another meta-analysis in 2010 and revealed that hyperuricemia was associated with a pooled risk ratio of 1.09 for CAD incidence. The subgroup analysis indicated that hyperuricemia increased the risk of CAD by 67% in women but not in men. Whether serum UA is a risk factor for CAD remains unclear. There is a lack of strong clinical evidence clarifying whether hyperuricemia treatment leads to improved CV outcomes. Animal studies of allopurinol use limited the infarct size (21) and enhanced recovery from the stunned myocardium (22). Noman et al. conducted a randomized trial and reported that allopurinol use for 6 weeks prolonged the time to angina in patients with chronic stable angina (23). However, Parmley et al. conducted another double-blinded, randomized study of 140 patients with ischemic heart disease, in which allopurinol increased the incidence of infarct extension (24). Nevertheless, these were all animal or small-sized clinical studies.

Singh et al. conducted a large series retrospective cohort study and reported that current allopurinol use, as opposed to prior allopurinol use, could protect patients with gout and diabetes against the occurrence of incident myocardial infarction or stroke (25). Unfortunately, the individual risks of new-onset CAD and stroke due to current allopurinol use were not provided in the study. To the best of our knowledge, our study is the first to report the potential protective effect of uricosuric agents against the development of CAD. The effect of uricosuric agents on the reduction of CAD appeared to exhibit a dose-response trend, which suggested that the decreasing risk of incident CAD depended on the increasing UA excretion of the kidney, not on a reduction in oxidative stress and inflammation. Savarese et al. (26) conducted a meta-analysis to evaluate the reduction of serum UA through pharmacologic therapies and its relationship with major CV events. They discovered no association between the reduction of serum UA and CV events. However, their study population was mostly composed of patients with asymptomatic hyperuricemia, which differs from our study population with gouty arthritis. Moreover, their endpoints were prevalent CV events whereas ours were incident CV diseases. Additionally, our study revealed that the cumulative incidences of incident CAD (Figure 2A) of with or without ULT tend to overlap after 10 years' follow up; which may indicate that ULT attenuates its protective effect for CAD development after long-term therapy. Studies disclosed that serum uric acid had stronger relationship with younger patients and those with fewer cardiovascular diseases (27). The strength of the association between uric acid and hypertension decreased with growing patient age and the duration of hypertension (28), implying that uric acid may be more important in younger patients with early-onset cardiovascular disease.

There are fewer reports of the effect of hyperuricemia on stroke than of that on CAD. Kim et al. conducted the only meta-analysis including 16 prospective cohort studies and revealed that hyperuricemia was associated with a moderate but significant higher risk of stroke incidence and mortality (9). A high serum UA level was demonstrated to have a linear relationship with carotid atherosclerosis (29). HT is a major contributor of stroke, and Soletsky et al. demonstrated that the use of ULT could lower blood pressure in obese adolescents with prehypertension (30). ULT might mitigate the risk of stroke incidence. In their cohort study, Singh et al. (25) revealed that current allopurinol use could reduce the risk of composite incident myocardial infarction or stroke. Our study also demonstrated that ULT could lower the risk of new-onset stroke. The protective effect of ULT was more prominent in patients taking uricosuric agents, male patients, and patients with HT. In our patients taking ACE inhibitors, ARBs, β-blockers, calcium channel blockers, or diuretics, ULT could still significantly lower the risk of stroke. UA may play an independent role in the development of stroke. However, the dose-response effects of ULT on CAD and stroke were different in our study. There was no significant doe response trend of cumulative mean DDD of uricosuric agents on the risk of incident stroke, we think this difference may be due to: (1) the event rate of stroke was less than that of CAD in our study. (2) More studies disclosed the association of serum uric acid with CAD than with stroke. (3) Serum uric acid has stronger impact on younger patients with fewer cardiovascular diseases, whereas stroke often occurs at older age. Most patients with stroke have comorbid hypertension or atrial fibrillation. Once patients have hypertension, the impact of serum uric acid on cardiovascular outcomes is attenuated (28).

Recent epidemiological studies have highlighted that we are confronted with an epidemic development of HF and its subsequent burden. UA is a parameter with strong prognostic significance over a wide range of HF severity levels. Hung et al. conducted a meta-analysis and reported that hyperuricemia was associated with a higher risk of incident HF and adverse outcomes in patients with HF (10). Xanthine oxidase inhibition has been suggested to improve various surrogate markers in patients with HF (31). No known study addressed the intervention of ULT and the development of HF. Our study indicated that the use of ULT had a neutral effect on the risk of incident HF; however, the event rates were low, which might be due to the young age of our population and the limited number of comorbidities.

The strengths and limitations of our study warrant discussion. First, randomized clinical trials with a long follow-up period are not easy to perform and not available in the literature to answer our study questions. We conducted a large series cohort study with an inception free of CV disease to observe the incidences of CAD, stoke, and HF among patients who received ULT and those who did not. These patients were propensity-score matched according to 30 clinical variables to maximally balance the possible confounders. But note that there are still some residual confounding factors that cannot be excluded. Second, the administrative database used did not contain information regarding the smoking status, alcohol drinking, physical activity, and body mass index, these unmeasured confounders may affect our censored outcomes. Third, we did not have access to the results of blood pressure or to those of biochemical blood tests, such as UA, glucose, cholesterol, and creatinine, which might have influenced the development of CV disease. Fourth, because our disease definition is based on codified data, misclassification bias may exit. Patients with some forms of arthritis may be misdiagnosed as gout without confirmation of uric acid or crystal identification. Fifth, in this study patients receiving ULT had longer follow-up time than patients not receiving ULT; it may indicate that patients taking ULT have healthy behavior with better adherence to treatment and overall prognosis. These patients may therefore have lower incidence of cardiovascular events. Sixth, because we recruited patients with gout, our results cannot be applied to hyperuricemic patients without gouty attack. Additionally, patients with a greater disease severity (such as higher uricemia or recurrent gouty attack) may be more likely to take ULT. This confounding by indication also requires attention. Finally, this was a hypothesis-driven study, in which some unknown biases certainly existed. A randomized control study would be necessary to verify our results.

## Conclusions

CAD and stroke are the leading causes of death worldwide. We encourage patients to aggressively treat their hyperuricemia to maximally mitigate the development of CV disease in the future.

## Data Availability Statement

National Health Insurance Research Database (NHIRD) published by Taiwan National Health Insurance (NHI) Bureau. The data utilized in this study cannot be made available in the paper, the supplemental files, or in a public repository due to the Personal Information Protection Act executed by Taiwan's government, starting from 2012. Requests for data can be sent as a formal proposal to the NHIRD (http://nhird.nhri.org.tw) or by email to nhird@nhri.org.tw.

## Ethics Statement

The studies involving human participants were reviewed and approved by the Research Ethics Committee of China Medical University and Hospital (CMUH-104-REC2-115). Written informed consent for participation was not required for this study in accordance with the national legislation and the institutional requirements.

## Author Contributions

F-SY and JW participated in the study concept and design. JW and H-LL participated in the acquisition of data. C-CH and H-LL participated in analysis and interpretation of data. F-SY and C-MH participated in drafting of the manuscript. F-SY, C-CH, JW, and C-MH participated in critical revision of the manuscript for important intellectual content. C-CH and H-LL participated in the statistical analysis. C-CH and JW participated in administrative, technical, or material support. JW and C-MH participated in study supervision.

### Conflict of Interest

The authors declare that the research was conducted in the absence of any commercial or financial relationships that could be construed as a potential conflict of interest.

## Abbreviations

ULT: urate-lowering therapy
CV: cardiovascular
CAD: coronary artery disease
ACE: angiotensin-converting enzyme
ARBs: angiotensin II receptor blockers
DDD: defined daily dose
SMD: standardized mean difference
SD: standard deviation
UA: uric acid.
